# Editorial Notes, News and Miscellany

**Published:** 1912-08

**Authors:** 


### Editorial Notes, News and Miscellany.

---

THINK OF IT, ye dwellers in the bleak places: cantaloupes 10 cents per dozen, big watermelons two for a quarter. Elberta peaches 50 cents per bushel at Austin.

---

*American Medicine's* gold medal has been awarded to Dr. W. C. Gorgas as "the American physician who has performed the most conspicuous and noteworthy service in the domain of medicine during the past year."

---

THE GOVERNOR decapitated Dr. J. D. Osborn, President Texas State Board of Medical Examiners, because he was a "Ramsey man," and appointed his son, Dr. E. B. Osborn, a member, because he was a "Colquitt man."

---

THE CONVENTION of North Dakota Homeopathic physicians urged that the Legislature pass a law compelling the asexualizing of all men who are found to be insane or whose criminal record stamps them as habitual criminals and as likely to be unfit for parenthood.

---

DEATH OF DR. CAFFERY.—It is with much regret we announce the death of Dr. Russell Caffery, of San Antonio. He died July 11th at his home in that city of a brief illness, at the early age of forty-five years. He was one of the foremost, most popular, most active and successful surgeons in a city famous for its surgeons. He was a Tulane man. Data for a fuller notice was received too late to print. D.

---

DRS. PETTEY & WALLACE'S SANITARIUM—CHANGE OF ADDRESS.—In systematizing street names, the name of the street on which Drs. Pettey & Wallace's Sanitarium is located has been changed from South Fourth Street to South Fifth Street. Please bear in mind that this change of address does not involve a change of location of the institution. Their new address is 958 South Fifth Street, Memphis, Tenn.

---

TO MAKE A FATAL PROGNOSIS is a risky thing. Your ability, however, to relieve pain if demanded, or to increase comfort, is always possible; and more than that. Many a "doomed" case of pulmonary edema, from heart disease, acute pneumonia, or nephritis gives the lie to a fatal prognosis, if you know how to utilize powerful and repeated subcutaneous doses of the double salt of caffein, or a few big doses (10 to 15 minims) of a good fluid extract of digitalis, or strophanthin intravenously with or without good hypodermic doses of camphor in sweet almond oil, or big doses of musk or sufficient doses of a nitrite. Only those of us who do not know what medicines can do deny their efficacy.—*Jacobi.*

---

GOVERNOR DIX SIGNS THE NEW YORK STERILIZATION BILL.—The bill providing for the sterilization of criminals and defectives, which was introduced by Dr. R. P. Bush, from Chemung county, and which was passed by both houses, was signed by Governor Dix. This law has been urged in order to reduce the number of children with inherited tendencies toward insanity and crime. New York is the sixth State in the Union to adopt this law.

The section appears under the Public Service Law, and is entitled, Operations for the Prevention of Procreation. The Governor is empowered to appoint one surgeon, one neurologist and one practitioner of medicine, each with at least ten years experience, to be known as the Board of Examiners for Feeble-Minded, Criminals and Other Defectives.—*Medical Fortnightly.*

---

TEXANS AT THE ATLANTIC MEETING, A. M. A.—An Association of State Secretaries and Editors was created, and our esteemed friend, Dr. Holman Taylor, Secretary Texas State Medical Association and editor of its *Journal,* was elected President. A new section was created (on hospitals), and Dr. W. B. Russ, of San Antonio, was made Chairman. A Lecture Bureau for each State was created, and Drs. C. E. Cantrell, I. C. Chase and M. M. Carrick were appointed to open the campaign in Texas. Our strenuous friend, Cantrell, was something like the Georgia militia captain who told his men to fire and fall back, and as he was a little lame he'd start at once. Dr. C. opened the ball in a negro church at Atlantic City Sunday night with an eloquent lecture to the darkies.

---

"THERE IS A REASON for the popularity of the proprietaries. Whether many of these were 'wonderful discoveries' or not, they have enabled the average physician to secure results more satisfactory to himself and his patients than he was able to secure without them. Very, very few medical men are able to extemporize prescriptions which at the same time are effective, palatable and not uselessly poly-pharmacal. All doctors ought to be able to do this, but they are not—and whose fault is it? And even if they were, who but the sheerest crank would claim that he could properly write for, or the average druggist dispense, substitutes as elegant, as cheap and withal so satisfactory as many of the best type of the proprietaries? It is best to look all these facts squarely in the face and be sensible in our conclusions."—*Southern Practitioner.*

---

IN VIEW OF THE APPALLING FACTS of the transmission of crime, epilepsy, and feeble-mindedness from generation to generation, the New Jersey Legislature at the session of 1910-11 passed a bill which empowered the Governor to appoint a commission to act in all cases recommended by the heads of the various institutions, the persons having the right of appeal to the court to show why the operation should not be performed. The law permits and specifies the operation—orchidectomy in rapists, and vasectomy in all other defectives. In the Indiana State Reformatory vasectomy has been performed in several hundred cases, and while it prevents procreation its does not destroy sexual desire or the ability for coition. The sterilization of defectives and delinquents is one of the newer questions coming before the profession and it deserves careful study and earnest consideration.—*New York Medical Journal.*

---

Colored Medical Students.—"While the learned professions are assuredly not the place to draw the lines of color or race, our experience compels us to disapprove of mixed classes. The negro, sitting in a white class, imagines he is looked upon askance by his classmate and suspicion is sure to engender this feeling. This detracts from that close attention the study of medicine requires and the man is unable to do himself justice.

"For this reason, and since there are well appointed colleges for colored medical students alone, we advise the latter to resort to their own schools."

This statement will appear in the forthcoming "Announcement" of Bennett Medical College, Loyola University Medical Department, of Chicago, Ill., and which we most heartily endorse and concur in. There is a very good medical school for colored students in Washington City, and Meharry Medical College of this city offers most excellent advantages and has been most favorably mentioned in "The Carnegie Foundation." (See ad. under "Contents.")—*Southern Practitioner.*

---

*Collier's Weekly,* for June 22d, has articles on "Two Patent Medicine Statesmen," referring to Champ Clark and Wm. Randolph Hearst. Concerning the former they quote a patent medicine advertisement from the *Garland News,* of Garland, Texas, which reads as follows: "Holds up a Congressman. 'At the end of the last campaign,' writes Champ Clark, Missouri's brilliant Congressman, 'from overwork, nervous tension, loss of sleep and constant speaking, I had about utterly collapsed. It seemed that all the organs in my body were out of order, but three bottles of Electric Bitters made me all right. It's the best all-round medicine ever sold over a druggist's counter! Overworked, run-down men and weak, sickly women gain splendid health and vitality from Electric Bitters. Try them. Only 50 cents. Guaranteed by Handly, druggist." As for Hearst, he is given a full page in which his portrait is surrounded by fac-similes of thirty-four of the most scandalous and fraudulent advertisements of as many quacks and fake medicines and appliances taken from his New York newspapers.

---

To Cut Off the Supply.—The people are becoming alarmed at the flood of degenerates coming into the world from degenerate parents, and the States of Indiana, California, Connecticut, Utah, Oregon, Iowa and New Jersey, and very recently New York, have passed laws providing for the sterilization of incurable lunatics and hopeless criminals; while the State Medical Associations of Florida and Louisiana have petitioned the Legislatures of those States for similar laws. Even the Homeopathic Association of North Dakota has fallen into line and made a similar petition to their State Legislature.

In Texas no action has been taken as yet by the State Association, but the editor of this Journal has been pegging away at it several years. At the last Legislature he had presented, by Dr. Daniel Parker, representative from Robertson county, a bill in line with those of other States; limited, however, to lunatics, and it passed the House, but hung fire in the Senate and died on the calendar—not reached because of the unseemly squabble about Jake Wolters and prohibition. Dr. Parker will have charge of the bill again at the coming session of the Legislature, and it will doubtless be backed by the State Association's committee on Legislation, and we have high hopes of success.

